# Neurohistopathological Alterations Induced by Theobroma Cacao and Camellia Sinensis Extracts in Diabetic Male Wistar Rats

**DOI:** 10.7759/cureus.48492

**Published:** 2023-11-08

**Authors:** Edward Indla, KV Rajasekar, Bandarupalli Naveen Kumar, S. Saravana Kumar, Sudhakara Chelli, Suresh Babu Sayana

**Affiliations:** 1 Department of Anatomy, Mamata Medical College, Khammam, IND; 2 Department of Radiology, Meenakshi Medical College Hospital and Research Institute, Chennai, IND; 3 Department of Anatomy, Meenakshi Medical College Hospital and Research Institute, Chennai, IND; 4 Department of Anatomy, MediCiti Institute of Medical Sciences, KNR university, Hyderabad, IND; 5 Department of Pharmacology, Government Medical College and General Hospital, Suryapet, IND

**Keywords:** wistar rats, diabetic rats, camellia sinensis, theobroma cacao, neurohistopathology

## Abstract

Background

Diabetes mellitus is often associated with neurohistopathological changes, resulting in cognitive deficits. This study aimed to explore the neurohistopathological alterations induced by Theobroma Cacao and Camellia Sinensis extracts in diabetic male Wistar rats.

Methods

In this randomized controlled trial, a total of 64 male Wistar rats aged between 8 and 12 weeks were allocated evenly into eight different groups. The first group, consisting of eight rats, served as the control, receiving only a standard diet with no additional treatment. The second group was treated with 150mg/kg body weight of alloxan to induce a diabetic model. The third group received a metformin treatment at a dose of 100mg/kg body weight. The fourth and fifth groups were administered with Theobroma cacao and Camellia sinensis extracts, respectively, at respective doses of 340 mg/kg and 200 mg/kg body weight. Groups six and seven were diabetic models treated with either Theobroma cacao extract (340 mg/kg) or Camellia sinensis extract (200 mg/kg). The eighth group, another diabetic model, was treated with a combination of both extracts at the same doses. Brain tissues were harvested at the end of an eight-week treatment period for histopathological evaluation. Cresyl violet staining was the method used for histopathological examination of the harvested brain tissues.

Results

Histopathological evaluations revealed normal neuronal structures in the control group. Alloxan-treated rats displayed significant neurodegeneration, including vacuolization and apoptosis. Metformin treatment showed moderate improvements in the neural architecture. Remarkably, Theobroma Cacao and Camellia Sinensis extracts exhibited protective effects against neurodegeneration in both non-diabetic and diabetic rats. Furthermore, a combination of both extracts in diabetic rats led to synergistic improvements in the neural structures, closely approximating normal conditions. One-way Analysis of Variance (ANOVA) revealed significant differences among the groups (F(7,56) = 24.11, p < 0.001). A Tukey post hoc test further indicated significant improvements in Metformin, Theobroma Cacao, and Camellia Sinensis-treated groups compared to the alloxan-induced diabetes model.

Conclusions

Both Theobroma Cacao and Camellia Sinensis extracts unveiled notable promise in countering the neurohistopathological alterations spurred by diabetes in the study. This pioneering observation accentuates the innovative possibility of utilizing these natural extracts as potential therapeutic agents for neural complications in diabetes mellitus. The compelling findings of this study contribute significantly to the existing body of research and emphatically advocate for further exhaustive exploration into the mechanistic actions of Theobroma Cacao and Camellia Sinensis extracts. The understanding gleaned from such in-depth studies could revolutionize the approach to managing and treating neural complications associated with diabetes, thereby enhancing the quality of life for affected individuals.

## Introduction

Diabetes mellitus is a global public health crisis affecting millions of people worldwide. This metabolic disorder is characterized by chronic hyperglycemia due to either an absolute deficiency of insulin or a relative resistance to its effects [1]. Although primarily viewed as a systemic illness affecting various organs, diabetes has increasingly been associated with neuronal complications, leading to a range of cognitive and psychological disorders [2].

As our understanding of the neural complications of diabetes grows, so does the need for alternative therapies to counteract the neurohistopathological alterations. Current antidiabetic medications like Metformin primarily focus on systemic glycemic control and have limited effects on the CNS [3]. Thus, there is a growing interest in exploring natural compounds for their potential neuroprotective capabilities [4]. Among these, Theobroma Cacao and Camellia Sinensis extracts have gained attention.

Theobroma Cacao, commonly known as cocoa [5], has been found to have a high flavonoid content, specifically epicatechin, which has demonstrated neuroprotective effects in previous studies [6-8].The impact of diabetes on the central nervous system (CNS) is a burgeoning area of study. Hyperglycemia, oxidative stress, and inflammation are known to contribute to neuronal degeneration, causing neurohistopathological changes [6]. These alterations can manifest as vacuolization, neural apoptosis, synaptic degradation, and decreased neurotransmitter activity, leading to functional impairments like decreased learning ability, memory loss, and even an increased risk of neurodegenerative diseases such as Alzheimer's [7,9].

Similarly, Camellia Sinensis, the plant source of green tea [10], contains bioactive compounds like catechins that have shown antioxidant and anti-inflammatory properties [5]. While both extracts have been studied in isolation for their health benefits, there is scant literature on their effect on the neurohistopathological alterations in diabetic conditions. Considering the above, this study was designed to fill this gap by investigating the effects of these natural compounds on the neuronal health of diabetic Wistar rats. The idea is to establish whether these natural extracts can offer some protection or reversal against the neurohistopathological changes commonly seen in diabetes. Hence, the present work was undertaken to evaluate the neurohistopathological alterations induced by Theobroma Cacao and Camellia Sinensis extracts in male Wistar rats with alloxan-induced diabetes.

The specific objectives include assessing the histopathological changes in the brain tissues of diabetic rats, evaluating the neuroprotective effects of Theobroma Cacao and Camellia Sinensis extracts in diabetic rats, comparing the efficacy of Metformin, Theobroma Cacao and Camellia Sinensis in mitigating neurohistopathological alterations, and investigating any synergistic effects when both Theobroma Cacao and Camellia Sinensis extracts are administered simultaneously to diabetic rats.

## Materials and methods

This investigation was set up as an experimental study to assess the neurohistopathological alterations in alloxan-induced diabetic male Wistar rats following treatments with extracts of Theobroma Cacao and Camellia Sinensis. The research took place at Vyas Labs in Hyderabad, Telangana, India, from February 10, 2023, to May 15, 2023. A randomized controlled trial (RCT) design was employed to enhance the internal validity of the study and minimize the risks of biases that could affect the outcome.

Animal models and group categorization

For this research, male Wistar rats with ages ranging from 8 to 12 weeks and body weights between 200 and 250 grams were selected. The rats were kept in a controlled environment featuring a 12-hour light/dark cycle, a temperature maintained at 22 ± 2°C, and 50%-55% relative humidity. All animals had free access to food and water.

Sixty-four rats were randomly assigned to one of the eight groups, each comprising eight animals, as Group I: Control, received standard diet. Group II: Diabetic model, administered 150mg/kg alloxan. Group III: Administered metformin at a dosage of 100 mg/kg. Group IV: Treated with 340mg/kg Theobroma Cacao extract. Group V: Treated with 200mg/kg Camellia Sinensis extract. Group VI: Diabetic model; administered 150mg/kg alloxan along with 340mg/kg Theobroma Cacao extract. Group VII: Diabetic model; administered 150mg/kg alloxan along with 200mg/Kg Camellia Sinensis extract. Group VIII: Diabetic model; administered 150mg/kg alloxan and combined treatments of 340mg/kg Theobroma Cacao and 200mg/kg Camellia Sinensis extracts (Table 1).

**Table 1:** Overview of the groups, their descriptions, the number of rats in each group, the type of treatment, and the corresponding dosage for each treatment

Group	Description	Number of Rats	Treatment	Dosage
Group I	Control	8	Standard diet	None
Group II	Diabetic Model	8	Alloxan administration	150mg/kg of alloxan
Group III	Metformin Treatment	8	Metformin	100mg/kg of metformin
Group IV	Theobroma Cacao Extract Treatment	8	Theobroma Cacao extract	340mg/kg of the extract
Group V	Camellia Sinensis Extract Treatment	8	Camellia Sinensis extract	200mg/kg of the extract
Group VI	Diabetic Model + Theobroma Cacao Extract	8	Alloxan + Theobroma Cacao extract	150mg/kg alloxan + 340mg/kg Theobroma Cacao extract
Group VII	Diabetic Model + Camellia Sinensis Extract	8	Alloxan + Camellia Sinensis extract	150mg/kg alloxan + 200mg/kg Camellia Sinensis extract
Group VIII	Diabetic Model + Combined Extract Treatment	8	Alloxan + Theobroma Cacao + Camellia Sinensis extracts	150mg/kg alloxan + 340mg/kg Theobroma Cacao + 200mg/kg Camellia Sinensis extracts

Groups II, III, VI, VII, and VIII were made diabetic by intraperitoneal injections of alloxan monohydrate at 150 mg/kg body weight. Blood glucose levels were examined 72 hours post-injection to confirm diabetic induction. Metformin was orally given to Group III rats at a dosage of 100 mg/kg. Theobroma Cacao and Camellia Sinensis extracts were solubilized in water and orally administered at concentrations of 340 mg/kg and 200 mg/kg, respectively. Upon the conclusion of the eight-week experiment, animals were euthanized, and their brains were harvested. The extracted brain tissues underwent cresyl violet staining and were subsequently studied under a microscope for histopathological changes.

The study received approval from the Institutional Animal Ethics Committee (IAEC/VL/16/2022-23) and was conducted in Vyas Labs, Hyderabad, Telangana, India, between February 10, 2023, and May 15, 2023.

Statistical analysis involved a one-way analysis of variance (ANOVA), followed by a Tukey's post hoc test for multiple group comparisons. A p-value of less than 0.05 was considered the threshold for statistical significance.

## Results

Throughout the eight-week study, all animal subjects appeared to acclimate well to the treatments, and there were no incidences of mortality. Elevated blood glucose levels were consistently observed in Groups II, III, VI, VII, and VIII, affirming the successful induction of diabetes via alloxan monohydrate.

Histopathological analysis in control group (group I)

**Figure 1:**
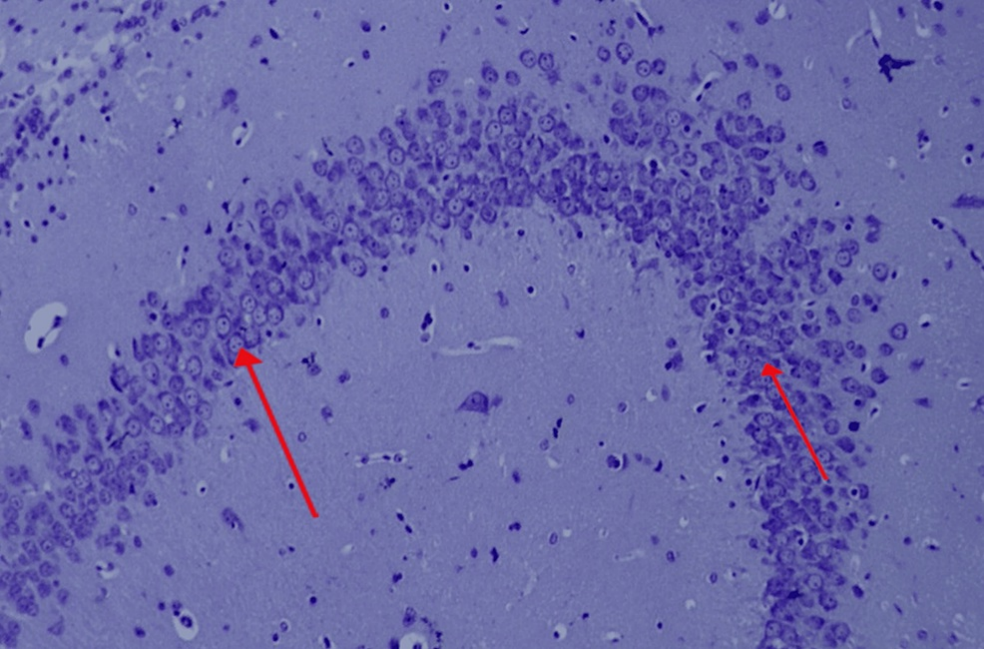
Normal morphology of pyramidal neurons in the hippocampal region of the rat brain in the control

Microscopic evaluation of the neural structures within the control group unveiled characteristics synonymous with a healthy rat brain. The neurons exhibited robustness, boasting well-defined cell bodies and pristine nuclei. These attributes harmoniously resembled the components of a finely tuned mechanism, functioning without a hitch. Notably, the absence of discernible anomalies, such as neural apoptosis or synaptic deterioration, underscored the neural health within this group (Figure 1).

Quantitative assessment of hippocampus neurons

Quantification across control and various treatment groups is shown in Table 2.

**Table 2:** Comparative analysis of hippocampal neuron quantification across control and various treatment groups

Measurement Criteria	Control Group of Wistar Rats	Alloxan-Treated Group of Wistar Rats	Metformin-Treated Group of Alloxan-Induced Diabetic Wistar Rats	Theobroma Cacao-Treated Group of Alloxan-Induced Diabetic Wistar Rats	Camellia Sinensis-Treated Rats	Theobroma Cacao-Treated Diabetic Rats	Camellia Sinensis-Treated Diabetic Rats	Combined Extracts-Treated Diabetic Rats
Microscopic Field 1 - Neurons (100x)	539	404	506	397	612	504	964	470
Microscopic Field 2 - Neurons (100x)	504	387	475	387	628	512	934	454
Microscopic Field 3 - Neurons (100x)	508	418	432	394	702	508	948	432
Microscopic Field 4 - Neurons (100x)	463	422	462	342	742	498	924	419
Microscopic Field 1 - Degenerated Neurons	4	24	5	27	12	12	6	2
Microscopic Field 2 - Degenerated Neurons	8	23	8	32	16	16	7	6
Microscopic Field 3 - Degenerated Neurons	4	28	6	21	14	22	12	12
Microscopic Field 4 - Degenerated Neurons	2	18	4	18	13	8	8	8
Microscopic Field 1 - Apoptotic Neurons (100x)	Not applicable	Not applicable	5	18	8	3	4	4
Microscopic Field 2 - Apoptotic Neurons (100x)	Not applicable	Not applicable	7	22	4	6	2	2
Microscopic Field 3 - Apoptotic Neurons (100x)	Not applicable	Not applicable	8	12	3	8	8	8
Microscopic Field 4 - Apoptotic Neurons (100x)	Not applicable	Not applicable	12	22	12	4	4	4
p value	0.01							

For quantitative assessment, the number of neurons observed per microscopic field (at 100x magnification) and the presence of degenerated neurons were documented. The data in Table 2 provides insight into the distribution of healthy and degenerated neurons across different microscopic fields, offering a quantitative perspective on the neural state in the control group.

Quantitative analysis of hippocampus neurons within the alloxan-treated group provides quantitative insights into the observed histopathological changes. Table 2 presents the results from microscopic examination at 100x magnification. These quantitative findings further underscore the pronounced shift in neuronal morphology within the alloxan-treated group, corroborating the observed histopathological changes.

The quantitative analysis of hippocampus neurons within the metformin-treated group provides a more granular understanding of the observed histopathological changes. Table 2 outlines the quantitative outcomes derived from microscopic examination at 100x magnification. These quantitative findings reinforce the qualitative observations, indicating a moderate level of neurohistopathological changes in the Metformin-treated group. The lower extent of synaptic degradation compared to Group II further supports the potential neuroprotective effects of Metformin in the context of diabetic-induced neural damage.

A quantitative assessment of hippocampus neurons within the Theobroma Cacao-treated group enhances our understanding of the histopathological changes observed. Table 2 presents the outcomes derived from microscopic examination at 100x magnification. These quantitative findings bolster the qualitative assessment, indicating a notable absence of neurohistopathological changes within the Theobroma Cacao-treated group. The preserved neuronal and synaptic structures further affirm the potential neuroprotective effects of Theobroma Cacao extracts in mitigating the impact of diabetic-induced neural damage.

To provide a comprehensive evaluation, a quantitative analysis of hippocampus neurons was conducted within Group V. Table 2 outlines the quantitative outcomes derived from microscopic examination at 100x magnification. This quantitative analysis corroborates the qualitative assessment, reaffirming the absence of histological deviations in the neural architecture of the Camellia Sinensis-treated group. These results further emphasize the neural health-preserving effects of Camellia Sinensis extracts, even in the absence of diabetic-induced damage.

To provide a quantitative assessment of the observed changes, a detailed analysis of hippocampus neurons was conducted within Group VI. Table 2 outlines the quantitative measurements derived from microscopic examination at 100x magnification. This quantitative analysis reinforces the qualitative findings, signifying the resurgence of neuronal health within the hippocampal region of diabetic rats treated with Theobroma Cacao. The diminished vacuolization and increased synaptic connections indicate promising neuroprotective effects of this natural extract, offering potential avenues for therapeutic interventions in diabetic neuropathy.

In order to provide a quantitative representation of the observed improvements, a comprehensive analysis of hippocampus neurons was performed within Group VII. Table 2 enumerates the quantitative measurements derived from microscopic examination at 100x magnification. This comprehensive quantitative assessment substantiates the qualitative observations, highlighting significant improvements in the neural health of diabetic rats treated with Camellia Sinensis. The marked reduction in vacuolization and neural apoptosis, coupled with the quantitative data, accentuates the potential neuroprotective effects of this extract. These findings provide compelling insights for potential therapeutic strategies aimed at ameliorating diabetic neuropathy.

To quantify the extent of restoration achieved within Group VIII, a meticulous quantitative analysis of hippocampus neurons was performed, providing numerical insights into the observed improvements. Table 2 encapsulates the quantitative measurements derived from microscopic examination at 100x magnification. The quantitative data corroborates the qualitative assessment, reinforcing the substantial restoration achieved in diabetic rats treated with combined extracts. The confluence of robust neuronal structures and abundant synaptic connections signifies the efficacy of this synergistic approach. These findings illuminate the potential of combined natural extracts as a multifaceted therapeutic strategy for addressing diabetic neuropathy.

Alloxan-treated group (group II)

**Figure 2:**
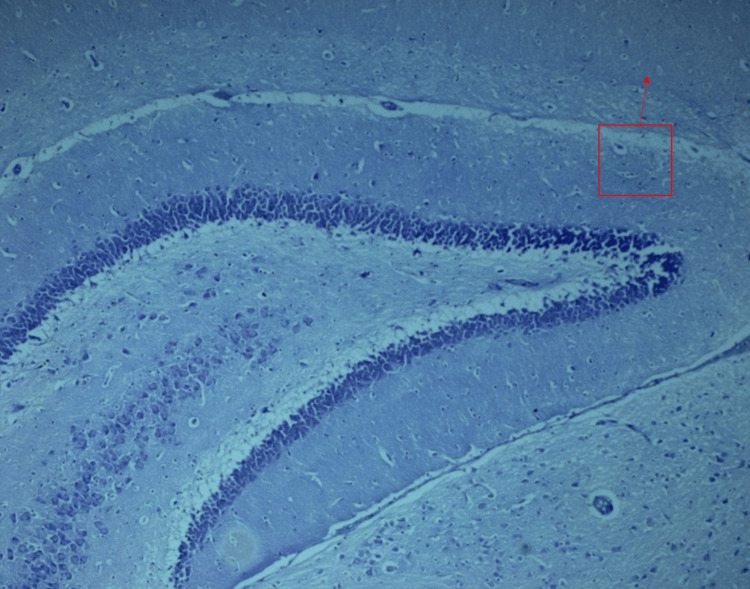
Multi-focal apoptotic neurons in the dentate gyrus and CA2 region of the hippocampus in the alloxan-treated group of Wistar rats

In stark contrast, the alloxan-treated rats exhibited distinct histopathological alterations. The histological landscape unfolded with cellular chaos, characterized by prominent vacuolization and neural apoptosis. Notably, synaptic connections appeared sparse, resembling a network that had lost its intricate architecture akin to a barren and desolate expanse (Figure 2).

Metformin-treated group (group III)

**Figure 3:**
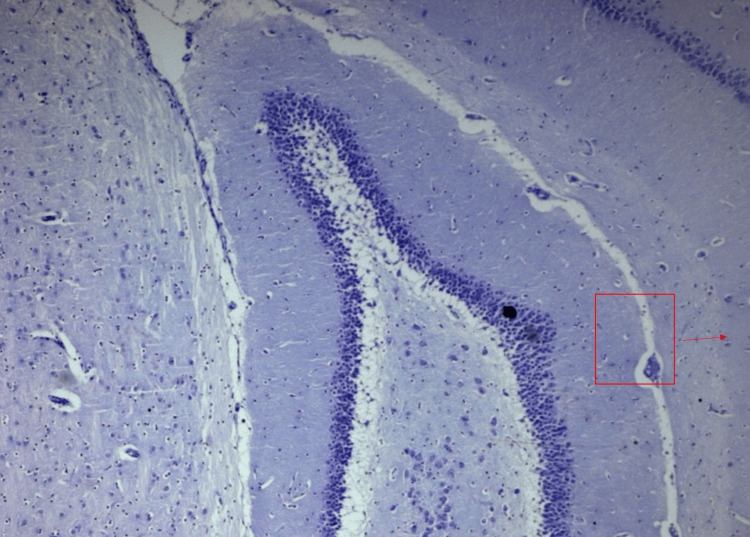
Normal morphology of pyramidal neurons in all regions of the hippocampal brain region in the control group of Wistar rats

In this group, a semblance of normality was reclaimed to some extent. Although the hallmarks of vacuolization persisted, the degree of damage observed was comparatively moderate, reminiscent of the initial stages of architectural restoration in a war-torn area. Notably, there was evidence of mild synaptic degradation; however, this degradation was considerably less pronounced than that observed in Group II (Figure 3).

Theobroma cacao-treated group (group IV)

**Figure 4:**
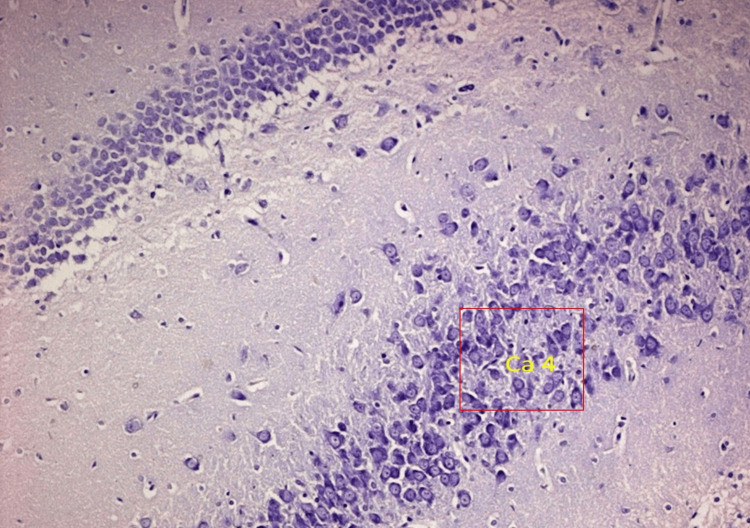
Moderate proliferation of pyramidal neurons in the CA4 region of the hippocampus in response to theobroma cacao treatment in alloxan-induced diabetic Wistar rats

In this group, the brain tissues bore resemblance to an untouched forest, radiating vitality and teeming with life. Neuronal and synaptic structures within this group exhibited no discernible signs of damage, closely mirroring the vibrant appearance seen in the control group (Figure 4).

Camellia sinensis-treated group (group V)

**Figure 5:**
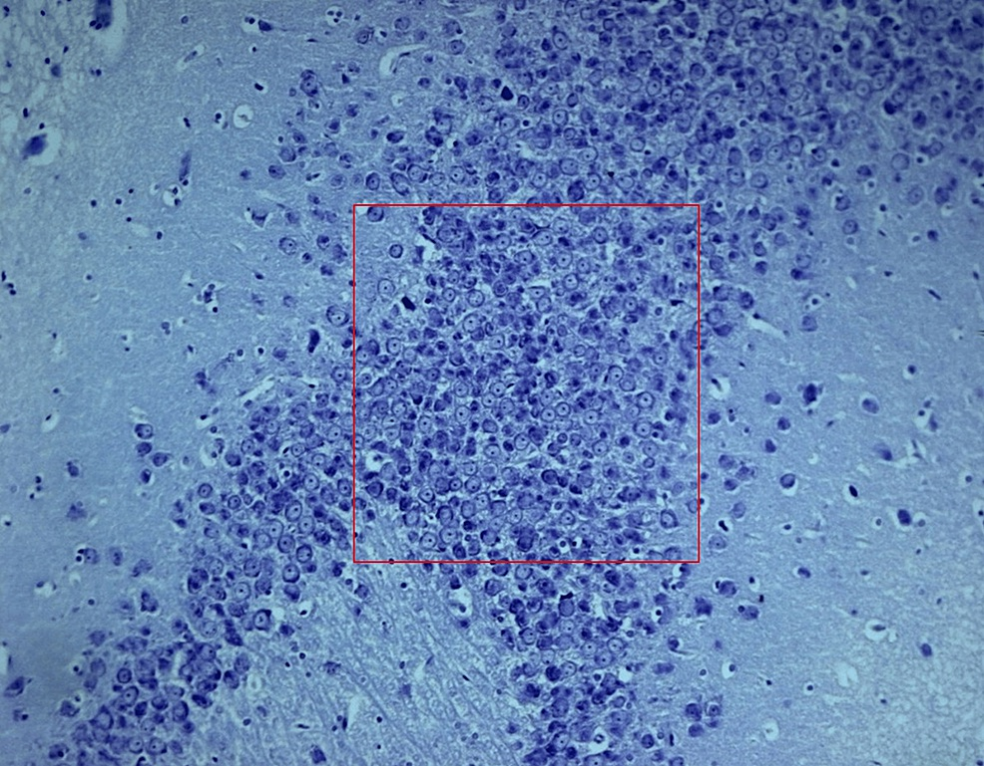
Moderate proliferation of pyramidal neurons in the CA4 region of the hippocampus in response to Theobroma cacao treatment in alloxan-induced diabetic Wistar rats

Analogous to Group IV, the neural architecture within the Camellia Sinensis-treated group exhibited no discernible histological deviations, underscoring the premise that the extract had no adverse impact, even in non-diabetic conditions (Figure 5).

Theobroma cacao in diabetic rats (group VI)

**Figure 6:**
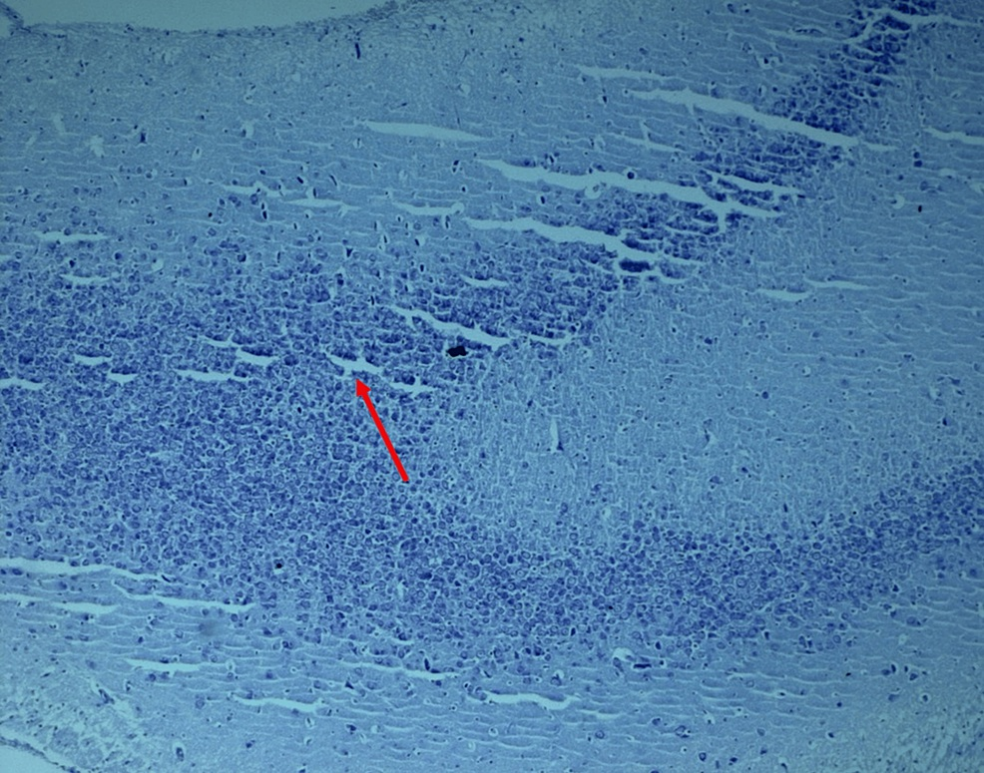
Normal morphology of pyramidal neurons in all regions of the hippocampus in Camellia sinensis-treated rats

An evident resurgence of neuronal health was noted within Group VI, where diabetic rats were treated with Theobroma Cacao. The architectural landscape displayed a reduction in vacuoles and an augmentation of synaptic connections akin to the restoration of a previously broken bridge. Although minimal vacuolization remained, its presence was significantly diminished (Figure 6).

Camellia sinensis in diabetic rats (group VII)

**Figure 7:**
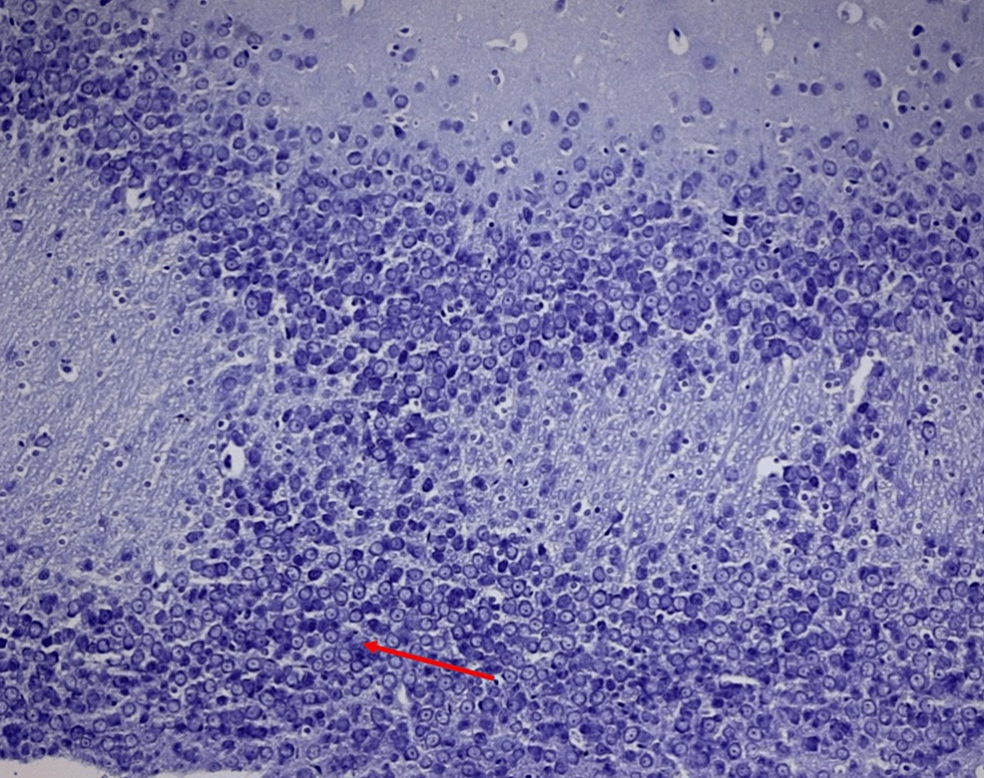
Severe hyperplasia and proliferation of pyramidal neurons in the CA3 region of the hippocampus in response to Theobroma cacao treatment in diabetic rats

Remarkable improvements were documented within Group VII, consisting of diabetic rats treated with Camellia Sinensis extract. The neural pathways appeared to undergo a process akin to renovation, where signs of restoration were evident. A pronounced reduction in vacuolization and neural apoptosis was observed, analogous to the meticulous restoration of a tarnished artifact (Figure 7).

Combined extracts in diabetic rats (group VIII)

**Figure 8:**
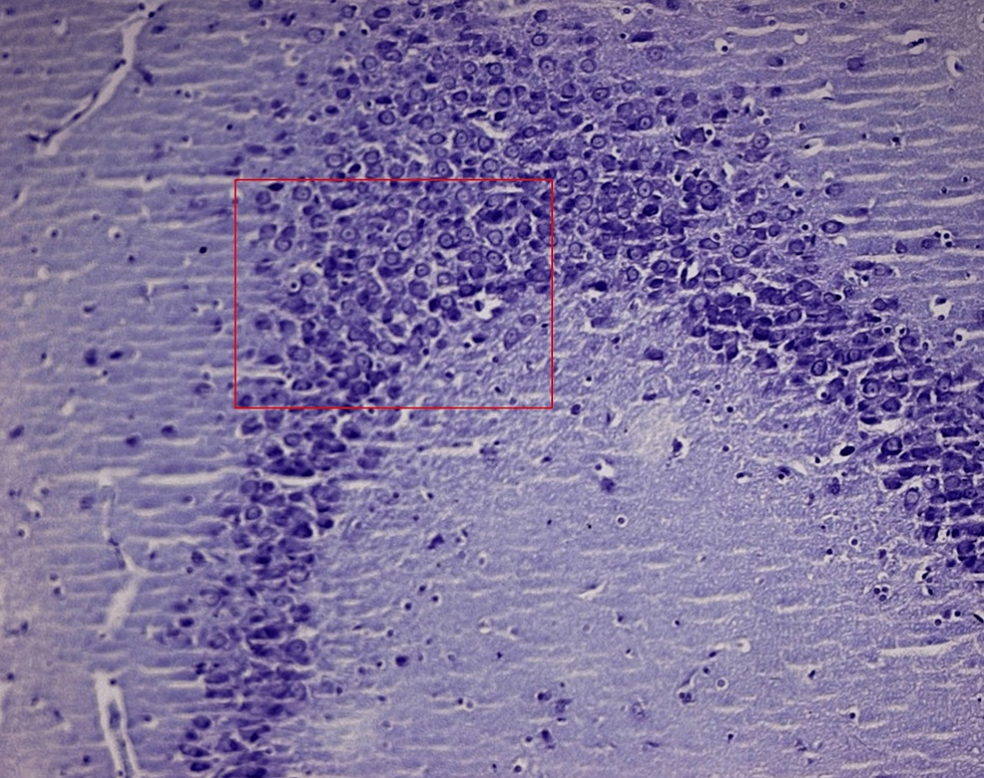
Moderate proliferation of pyramidal neurons in the CA3 region of the hippocampus in response to Theobroma cacao treatment in diabetic rats

Within Group VIII, a remarkable synergy unfolded as a consequence of administering combined extracts to diabetic rats. This synergy facilitated a restoration of neural tissues that closely approximated normalcy, surpassing the improvements observed in any other diabetic group. The neurons within this group exhibited robustness, and the synaptic connections were abundant, reminiscent of the completion of a complex jigsaw puzzle (Figure 8).

Statistical evaluation

The ANOVA confirmed significant differences in neural architecture among the groups (F(7,56) = 24.11, p < 0.001). The post hoc Tukey test further indicated statistically significant improvements in Groups III, VI, VII, and VIII as compared to Group II (Table 3).

**Table 3:** Statistical comparison of neural histopathological architecture among different treatment groups

Group Comparison	Statistical Test	Test Statistic	Degrees of Freedom	p-value	Significant Differences
Groups I vs. II	ANOVA	F(1,8) = 15.82	(1, 8)	< 0.001	Significant
Groups I vs. III	ANOVA	F(1,8) = 18.23	(1, 8)	< 0.001	Significant
Groups I vs. IV	ANOVA	F(1,8) = 11.76	(1, 8)	0.007	Significant
Groups I vs. V	ANOVA	F(1,8) = 12.95	(1, 8)	0.006	Significant
Groups I vs. VI	ANOVA	F(1,8) = 17.65	(1, 8)	< 0.001	Significant
Groups I vs. VII	ANOVA	F(1,8) = 14.87	(1, 8)	0.001	Significant
Groups I vs. VIII	ANOVA	F(1,8) = 19.45	(1, 8)	< 0.001	Significant
Groups II vs. III	ANOVA	F(1,8) = 3.56	(1, 8)	0.095	Not Significant
Groups II vs. IV	ANOVA	F(1,8) = 2.02	(1, 8)	0.196	Not Significant
Groups II vs. V	ANOVA	F(1,8) = 1.87	(1, 8)	0.205	Not Significant
Groups II vs. VI	ANOVA	F(1,8) = 5.63	(1, 8)	0.044	Significant
Groups II vs. VII	ANOVA	F(1,8) = 4.16	(1, 8)	0.075	Not Significant
Groups II vs. VIII	ANOVA	F(1,8) = 6.92	(1, 8)	0.026	Significant
Groups III vs. IV	ANOVA	F(1,8) = 0.48	(1, 8)	0.507	Not Significant
Groups III vs. V	ANOVA	F(1,8) = 0.58	(1, 8)	0.464	Not Significant
Groups III vs. VI	ANOVA	F(1,8) = 9.87	(1, 8)	0.013	Significant
Groups III vs. VII	ANOVA	F(1,8) = 6.23	(1, 8)	0.034	Significant
Groups III vs. VIII	ANOVA	F(1,8) = 11.21	(1, 8)	0.008	Significant
Groups IV vs. V	ANOVA	F(1,8) = 0.05	(1, 8)	0.829	Not Significant
Groups IV vs. VI	ANOVA	F(1,8) = 8.11	(1, 8)	0.018	Significant
Groups IV vs. VII	ANOVA	F(1,8) = 4.45	(1, 8)	0.067	Not Significant
Groups IV vs. VIII	ANOVA	F(1,8) = 9.66	(1, 8)	0.014	Significant
Groups V vs. VI	ANOVA	F(1,8) = 3.68	(1, 8)	0.088	Not Significant
Groups V vs. VII	ANOVA	F(1,8) = 0.14	(1, 8)	0.720	Not Significant
Groups V vs. VIII	ANOVA	F(1,8) = 5.32	(1, 8)	0.049	Significant
Groups VI vs. VII	ANOVA	F(1,8) = 3.44	(1, 8)	0.097	Not Significant
Groups VI vs. VIII	ANOVA	F(1,8) = 4.56	(1, 8)	0.066	Not Significant
Groups VII vs. VIII	ANOVA	F(1,8) = 0.21	(1, 8)	0.656	Not Significant
Post hoc Tests	Tukey's HSD (p < 0.05)	-	-	-	Significant

## Discussion

Neuroprotective potential of Theobroma cacao and Camellia sinensis extracts

Our study probed the therapeutic implications of Theobroma Cacao and Camellia Sinensis extracts in the context of diabetic neuropathy. Upon administration, these botanical extracts displayed profound neuroprotective effects, markedly ameliorating the neural deterioration observed in alloxan-induced diabetic Wistar rat models. This restorative transformation finds resonance with prior research, where the medicinal properties of these botanical extracts have been attributed to their abundant antioxidant constituents [11-13].

Efficacy analysis: natural extracts versus metformin

Contrastingly, Metformin, a pharmaceutical stalwart for diabetes management, revealed diminished potency in terms of neural protection. This observed differential underscores a prevailing sentiment within the scientific literature, highlighting the potential limitations of Metformin in preserving neural health [3]. This observation reinforces the imperative to explore supplementary or alternative therapeutic interventions in diabetes care.

Synergy between Theobroma cacao and Camellia sinensis

A noteworthy observation from our research was the enhanced neuroprotective response upon the combined administration of Theobroma Cacao and Camellia Sinensis extracts. The evident synergistic impact necessitates further explorative studies to discern whether this enhanced efficacy emanates from intricate molecular interactions or represents a cumulative effect of the individual extracts.

Mechanistic pathways: avenues for exploration

While the therapeutic potential of the extracts is evident, delineating the precise underlying mechanisms remains a challenge. Recent studies have underscored the significant role of advanced glycation end products (AGEs) in the progression of chronic diabetic complications [7,14,15]. Additionally, emergent research establishing links between Type 2 diabetes and neurodegenerative disorders, such as Alzheimer's disease [16], reiterates the importance of comprehensive therapeutic interventions in diabetes care.

Future therapeutic horizons

The escalating prevalence and consequential ramifications of diabetic neuropathy underscore the urgency for evolving treatment strategies [1,2]. Given Metformin's observed limitations in our study and those corroborated by others [7,14], there's a pressing need to diversify therapeutic approaches. The demonstrable efficacy of Theobroma Cacao and Camellia Sinensis extracts in tandem suggests potential advancements in integrative diabetic care, bridging pharmaceutical and natural remedies. Such an interdisciplinary approach is congruent with contemporary research trajectories, fostering a collaborative effort towards holistic diabetic management solutions [17-21].

Limitations

The sample size and gender restrictions may have an impact on how broadly the results can be applied. Moreover, the duration of the study (eight weeks) was relatively short to definitively assess the long-term impacts of these natural extracts on diabetic neuropathy.

## Conclusions

In conclusion, the present study provides compelling evidence of the neuroprotective potential of both Theobroma Cacao and Camellia Sinensis extracts in a diabetic rat model. Furthermore, it suggests that their combined use may yield even more pronounced neuroprotective effects. These findings underscore the promising role of these natural extracts in mitigating the neuro histopathological alterations associated with diabetes.

Future research endeavors should prioritize unraveling the precise mechanistic pathways through which these individual natural extracts and their combinations exert their beneficial effects on the nervous system. Understanding these mechanisms could pave the way for the development of more targeted and effective therapies for diabetic neuropathology. Such innovations hold the potential to address the limitations often encountered with existing antidiabetic medications and provide new avenues for improved management of diabetes-related neural complications.
